# Autologous mesenchymal stromal cells embedded in tricalcium phosphate for posterolateral spinal fusion: results of a prospective phase I/II clinical trial with long-term follow-up

**DOI:** 10.1186/s13287-019-1166-4

**Published:** 2019-02-22

**Authors:** Juan F. Blanco, Eva M. Villarón, David Pescador, Carmen da Casa, Victoria Gómez, Alba M. Redondo, Olga López-Villar, Miriam López-Parra, Sandra Muntión, Fermín Sánchez-Guijo

**Affiliations:** 1 Trauma and Orthopedics Service, IBSAL – University Hospital of Salamanca, Salamanca, Spain; 2 Hematology Service, IBSAL – University Hospital of Salamanca, Salamanca, Spain; 3 Network Center in Regenerative Medicine and Cellular Therapy of Castilla y León, Salamanca, Spain; 4 Trauma and Orthopedics Department, IBSAL – University Hospital of Salamanca, Paseo de San Vicente 58-182, 37007 Salamanca, Spain

**Keywords:** Spine surgery, Autologous mesenchymal stromal cells, MSC, Spinal fusion, Lumbar degenerative disc disease, DDD, Bone graft, Cell therapy

## Abstract

**Background:**

Posterolateral spinal fusion with autologous bone graft is considered the “gold standard” for lumbar degenerative disc disease (DDD) when surgical treatment is indicated. The potential role of mesenchymal stromal cells (MSCs) to replace the bone graft in this setting has not been fully addressed.

**Objective:**

To analyze the safety, feasibility and potential clinical efficacy of the implantation of autologous MSCs embedded with tricalcium phosphate as a therapeutic alternative to bone graft in patients with DDD during posterolateral spine fusion.

**Study design:**

Phase I/II single-arm prospective clinical trial.

**Methods:**

Eleven patients with monosegmental DDD at L4–L5 or L5–S1 level were included. Autologous bone marrow-derived MSC were expanded in our Good Manufacturing Practice (GMP) Facility and implanted during spinal surgery embedded in a tricalcium phosphate carrier. Monitoring of patients included a postoperative period of 12 months with four visits (after the 1st, 3rd, 6th, and 12th month), with clinical and radiological assessment that included the visual analog scale (VAS), the Oswestry disability index (ODI), the Short-Form Health Survey (SF-36), the vertebral fusion grade observed through a simple Rx, and the evaluation of possible complications or adverse reactions. In addition, all patients were further followed up to 5 years for outcome.

**Results:**

Median age of patients included was 44 years (range 30–58 years), and male/female ratio was (6/5) L4–L5 and L5–S1 DDD was present five and six patients, respectively. Autologous MSCs were expanded in all cases. There were no adverse effects related to cell implantation. Regarding efficacy, both VAS and ODI scores improved after surgery. Radiologically, 80% of patients achieved lumbar fusion at the end of the follow-up. No adverse effects related to the procedure were recorded.

**Conclusions:**

The use of autologous MSCs for spine fusion in patients with monosegmental degenerative disc disease is feasible, safe, and potentially effective.

**Trial registration:**

no. EudraCT: 2010–018335-17; code Identifier: NCT01513694 (clinicaltrials.gov).

**Electronic supplementary material:**

The online version of this article (10.1186/s13287-019-1166-4) contains supplementary material, which is available to authorized users.

## Background

Lumbar degenerative disc disease (DDD) is a progressive and irreversible process that produces lumbar and/or radicular pain and is one of the most common causes of disability. Chronic low back pain is a serious health problem and originates an important healthcare cost. The etiology of this condition is multifactorial including the influence of certain genetic predispositions [1] and various risk factors such as load-bearing [2], autoimmune disorders [3], nutritional imbalances [4], molecular and cellular abnormalities [5] that lead to cellular loss, and dehydration of the disc, along with structural alterations compromising its biomechanics.

Established treatments are based on conservative methods such as physiotherapy, analgesia, or behavior modification. Surgical treatment may be indicated when conservative treatments have failed, and in this case, spinal fusion is the option most commonly used [6]. Spinal fusion procedures have greatly increased in the last few years [7]. The surgical procedure for spine fusion may involve the use of autograft or allograft bone tissue, and in some cases, bone substitutes are added [8]. The iliac crest autograft has demonstrated fusion rates higher than 90% in some cases [9] and can be an ideal material for spine fusion because of its osteogenic, osteoinductive, and osteoconductive ability. Nevertheless, this surgical procedure is not exempt from complications or inconveniences [10] such as donor site morbidity (acute or chronic pain and functional repercussions) and longer surgical time. Bone grafts retrieved from the surgical site may also be a good option and have demonstrated fusion rates similar to iliac crest grafts, but its main limitation is the availability of enough amount of graft. Allografts are used in different forms (freeze-dried, fresh-frozen, cancellous chips, and demineralized bone matrix), frequently combined with autograft, since it only has osteoconductive and mild osteinductive properties. Employed alone, there are controversial data on its efficacy. Since An et al. [11] showed low-fusion rates, other authors have demonstrated similar results to autografts, with less adverse events [12]. Other grafts and materials have also been tested. The use of calcium phosphate ceramics in its various forms has only osteoconductive capability and requires the addition of other elements, such as autograft or bone marrow aspirate. Fusion rates with ceramics alone were very low [13].

Therefore, interest in cell therapy has grown in recent years due to the ability of mesenchymal stromal cells (MSCs) to differentiate into various cell types. One of the most interesting ones is the potential of MSCs to produce or regenerate bone tissue [14]. Another attractive property of MSC is a strong immunomodulatory capacity [15]. MSCs can be obtained from various sources such as bone marrow (BM) from the iliac crest, abdominal fat, and others. These cells have been assessed clinically for the treatment of various diseases [16, 17].

Although the preclinical data on different animal models of spinal fusion has been extensively shown, there are few prospective clinical trials that have evaluated the role of MSC, expanded in a GMP facility, and released according to the International Society for Cellular Therapy (ISCT) criteria [18]. Therefore, it was mandatory by the Spanish regulators to start testing this product in a small prospective phase I/II trial with a maximum number of 15 patients. This was also a final part of a research line started by the in vitro characterization of the interactions of MSC with tricalcium phosphate (TCP) and their preclinical evaluation in a rabbit animal model of bone regeneration [19]. The trial was supported by a public grant from the National Health System (see Acknowledgements), and the median cost of the cellular product was around 6000€ per dose per patient.

The aim of this work was to assess the feasibility and safety of the use of autologous bone marrow-derived MSCs embedded in a tricalcium phosphate carrier in patients with monosegmental lumbar degenerative disc disease undergoing posterolateral spine fusion.

## Methods

An open, single-center, prospective, single-arm phase I/II clinical trial was performed with one experimental treatment group (EC Code: CSM/Fusion/2009 – EudraCT: 2010-018335-17; https:/clinicaltrials.gov code Identifier: NCT01513694). The trial was reviewed and approved by the Ethics Committee of the University Hospital of Salamanca and the Spanish Medicines Agency (AEMPS). All patients signed the approved informed consent, and all the procedures where in accordance to the principles of the Declaration of Helsinki.

### Study population

Fourteen patients were screened for inclusion into the trial (6 males and 8 females) of whom three were excluded (1 case of syphilis and 2 cases of viral hepatitis), thus resulting in a total of 11 patients surgically treated in our hospital between 2010 and 2013 finally included in the study. Inclusion criteria were capacity to consent, age between 18 and 65 years old, lumbar and/or radicular pain refractory to conservative treatment for more than 6 months, radiological diagnosis of monosegmental disc disease (L4–L5 or L5–S1), and grade IV or V of the Pfirmann scale on MRI [20]. Exclusion criteria included penicillin allergy; pregnant or breast-feeding women; those who suffered a genetic or acquired structural anomaly that contraindicated the procedure; had relevant co-morbidities (e.g., severe psychiatric diseases, cancer, inflammatory, or infectious diseases); those treated with prior chemotherapy, corticosteroid, or immunosuppressant drugs; and those that had received another experimental agent in the last 30 days. All patients complained of back and radicular pain. Patient’s details are summarized in Table 1.

**Table 1:** Patients’ characteristics

Patient	Age (years)	Gender	Back pain	Radicular pain	Level	Pfirmann	ASA
1	41	Male	Yes	Yes	L4-L5	V	I
2	30	Female	Yes	Yes	L4-L5	IV	I
3	52	Male	Yes	Yes	L4-l5	IV	II
4	50	Male	Yes	Yes	L5-S1	V	I
5	44	Male	Yes	Yes	L5-S1	V	II
6	54	Female	Yes	Yes	L4-l5	IV	II
7	42	Male	Yes	Yes	L5-S1	IV	I
8	33	Female	Yes	Yes	L5-S1	V	I
9	41	Female	Yes	Yes	L5-S1	V	I
10	49	Female	Yes	Yes	L4-L5	V	II
11	50	Female	Yes	Yes	L5-S1	IV	I

### Cell production and preparation

Cell production was performed in the GMP Cell Production Unit of the University Hospital of Salamanca, as previously described [21], with the slight modification of adding tricalcium phosphate (TCP) to the final MSCs product. The reference number of the Investigational Medicinal Product Dossier (IMPD) approved by the Spanish Medicines Agency (AEMPS) for cell production was PEI-10-007. Briefly, 40–100 mL of bone marrow (BM) was obtained from each patient’s iliac crest in the operating room following standard procedures and then transferred in sterile conditions to the GMP facility. Mononuclear cells were isolated after density-gradient centrifugation (Ficoll-Paque, GE Healthcare Biosicences, AB, Uppsala, Sweden); cultured initially at 160,000 cells/cm2 in an uncoated polystyrene surface (Corning Costar, Milan, Italy) with an expansion medium containing alpha-modified Eagle’s Medium (α-MEM) with 5% of platelet lysate (PL), 1% penicillin/streptomycin (Gibco, Paisley, UK), and 2 UI/ml heparin (Hospira, Alcobendas, Spain); and maintained at 37 °C in a 5% CO2 atmosphere. PL was obtained as previously reported. After 70–80% confluence, cells were detached with TrypLE Select (Gibco, Paisley, UK) and subcultured at 1000 cells/cm2. After two to three passages, cells were harvested and the final product containing 0.5–1.5 × 10^–6^ cells/kg from the patient in a sterile cell suspension was mixed in 20 mL with a tricalcium phosphate (TCP) support (Conduit-TC Granules 10 ml, Depuy-Spine, Raynham, MA, US). This final product was loaded in two syringes and taken to the operating room.

### Surgical procedure and cell administration

An instrumented posterolateral arthrodesis was performed in all cases, and the same surgical team performed all of the procedures. General anesthesia was used in all cases with antibiotic prophylaxis with cefazolin 2 g i.v. during anesthetic induction. A posterior middle-line approach was employed. Titanium pedicular screws were used, and the transverse process surfaces were decorticated by curettage. In one case, a left hemi-laminectomy and L5 radicular decompression was performed. Application of final product (MSCs and TCP mixture) into the intertransverse space, which is the fusion site, was performed in all cases. The content of each syringe was applied on one side. This final product was the only substance used for spinal fusion.

The wound was closed with vacuum aspiration drainage. All patients received thromboembolic prophylaxis with enoxaparin 40 mg s.c. per day during 10 days. At the first day after surgery, patients are allowed to be in a sitting position without the need of using lumbar orthosis and to ambulate after the second day. They were later discharged after a median of 5 days (range 4–6 days).

### Evaluation

Follow-up included at least a postoperative period of 12 months with four visits (at the 1st month, 3rd month, 6th month, and after 1 year) where the clinical and radiological outcomes were studied, in terms of spinal fusion observed in simple and lateral-stress radiography, and also, any adverse effect that occurred during the process was recorded. Additional clinical variables analyzed included (a) clinical efficacy: visual analog scale (VAS) [22], (b) functional disability: Oswestry disability index (ODI) [23], and (c) quality of life questionnaire: (SF-36) [24]. Radiological analysis was focused on the assessment of radiological fusion: existence of clear bone bridges in simple anteroposterior (AP), lateral views, and dynamic (flexion and extension) radiographs together with the absence of radiolucent lines (delayed consolidation or pseudo-arthrosis).

Although the established initial follow-up of the trial was 12 months, we followed all patients for another 5 years to assess safety and efficacy in the long term.

## Results

### Patients and cell product

As already mentioned, the final population consisted of a single experimental treatment group of 11 patients (5 males and 6 females) with a mean age of 44 years (range 30–55 years), all of them with clinical signs of lumbar or radicular pain. 54.5% had disc disease compromising L5–S1 and 45.5% compromising L4–L5 level. The 54.5% belonged to Pfirmann scale stage IV, and the other 44.5% were in stage V. The anesthetic risk on these patients as classified by the American Society of Anesthesiologist (ASA) score [25] was ASA 1 in seven patients and ASA 2 in four patients.

The main characteristics of the cellular product applied are summarized in Table 2.

**Table 2:** Characteristics of the cell product administered

Patient	Weight (Kg)	Dosage (× 10^6^ per Kg)	Phase	Days of expansion	Viability (% of viable cells)	Karyotype	In vitro differentiation
1	88	1.5	2°	25	95	46, XY [17]	Yes
2	82	1.5	1°	18	91,8	46, XX [22]	Yes
3	75.3	1.06	1°	18	92	No Metaphases	Yes
4	75	1.33	1°	26	97	46, XY [21]	Yes
5	94	1.5	1°	20	94	46, XY [20]	Yes
6	68	1.5	2°	26	97	46, XX [20]	Yes
7	98	1.5	1°	21	94	46, XY [20]	Yes
8	74	1.5	1°	20	95	No Metaphases	No
9	51	1.5	1°	21	94	46, XX [15]	Yes
10	84	1.5	2°	26	100	46, XX [20]	Yes
11	64	1.5	2°	21	95	46, XX [21]	Yes

### Clinical and functional results

The analysis of VAS, for both lumbar and radicular pain, showed that patients improved during the postoperative period compared to preoperative period. Radicular pain decreased more significantly than lumbar pain, demonstrated by the reduction from 8 to 3 and 8 to 5, respectively, at the final follow-up (1 year). Both lumbar and radicular pain improved significantly after 4 years of the surgical procedure (Table 3).

**Table 3:** VAS scores for radicular and lumbar pain

	Median	Minimum	Maximum
VAS basal, radicular	8	3	10
VAS 1 month, radicular	4.5	0	8
VAS 1 months, radicular	1.3	0	6.8
VAS 6 months, radicular	0.9	0	8.4
VAS 12 months, radicular	3.6	0	8.6
VAS basal, lumbar	8.2	5	10
VAS 1 month, lumbar	3.6	1.5	7.8
VAS 1 months, lumbar	4	4	6.6
VAS 6 months, lumbar	3	0.3	7.5
VAS 12 months, lumbar	5.1	0	8.8

Regarding the ODI analysis, patients also improved. The patients presented a severe functional limitation (basal ODI 56%) at the beginning of the treatment, whereas 12 months after MSC and surgical treatment, they displayed moderate functional limitation (ODI 31%) (Table 4).

**Table 4:** Values of ODI

	Median	Minimum	Maximum
ODI basal	56	12	74
ODI 1 month	32	20	66
ODI 3 months	29	8	60
ODI 6 months	22	6	60
ODI 12 months	31	4	62

The physical and mental status, evaluated by the SF-36 questionnaire, showed also a significant improvement the first year after surgery. In addition, ten of the 11 patients returned to work. One of them changed the type of work. The patient who did not return to the work activity was unemployed before the intervention and was involved in medico-legal litigation.

Patient number 2, which needed L5 root decompression during the surgical procedure, received further analgesia for episodes of low back pain during follow-up. The patient was receiving antidepressant treatment before the surgery, but he was not suffering from a serious mental illness. This patient, who at the time of the selection was unemployed, did not return to work at the end of the follow-up nor 5 years later.

### Radiological results

**Fig. 1:**
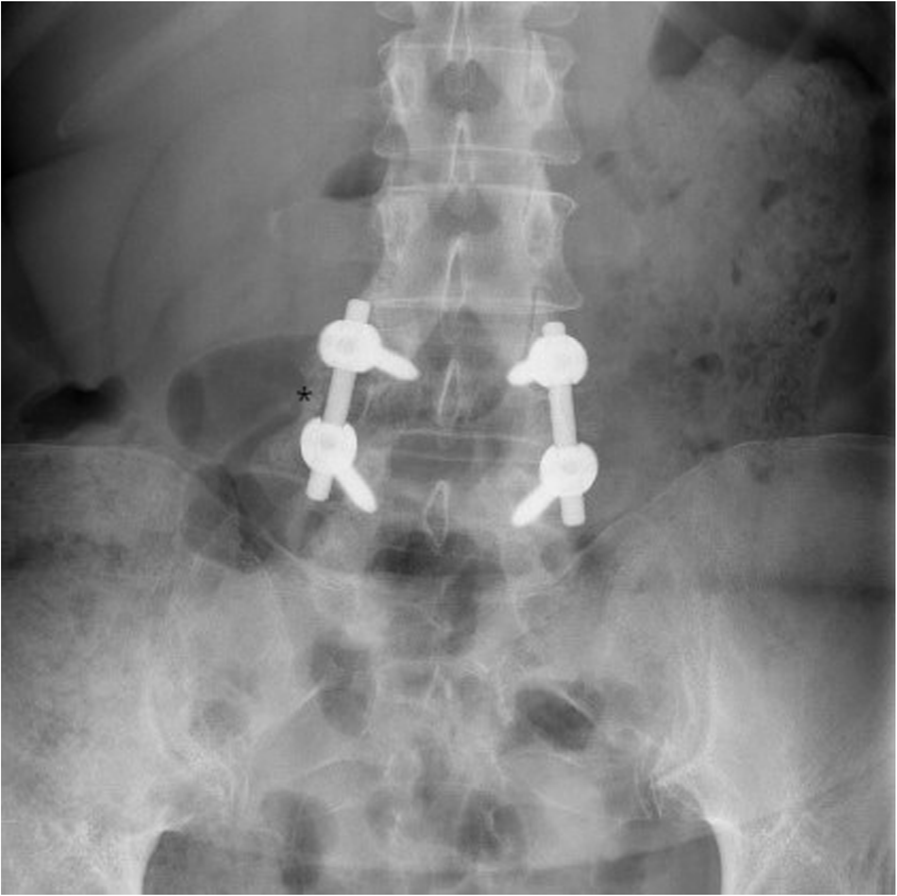
Anteroposterior X-ray of L4–L5 showing bone bridges (asterisk) formation in the intertransverse space for posterolateral spine fusion 1 year after surgery

**Fig. 2:**
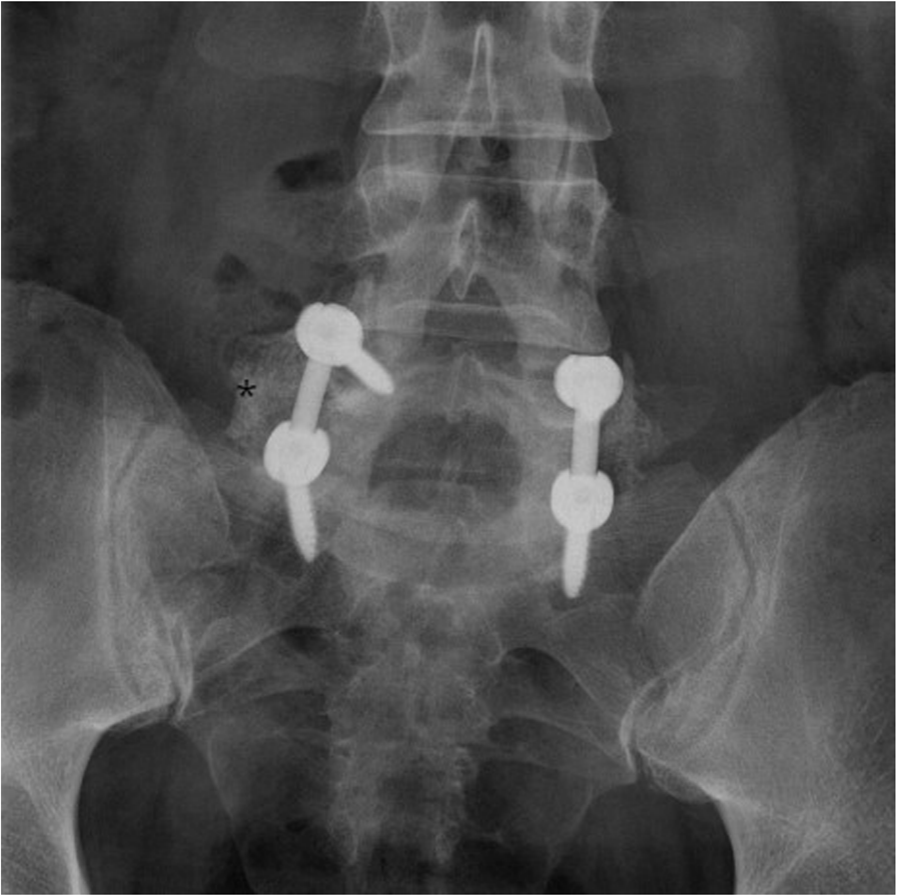
Anteroposterior X-ray of L5–S1 showing bone bridges (asterisk) formation in the intertransverse space for posterolateral spine fusion 1 year after surgery

Radiological solid fusion was successfully obtained in nine of 11 of the cases observed in simple Rx focused on the lumbar spine by the end of the study. No motion segments, lytic areas, or pseudo-arthrosis were detected in any case (Figs. 1 and 2).

### Complications and side effects

At the end of initial follow-up (1 year) as well as up to 5 years later, there were no complications or adverse side effects related to the procedure, including heterotopic ossification, infections, or tumors attributable to the MSC treatment administered.

## Discussion

Cell therapy applied to the spinal disorders has focused mainly in two areas: on the one hand, in intradiscal cell therapy for the treatment of degenerative disc disease, and on the other hand, on the use of cells to improve spinal fusion procedures [26]. Degenerative disc disease and its consequences, low back and radicular pain, are very common health problems. When conservative treatment fails, surgical treatment by spinal fusion is usually indicated. Currently, spinal fusion is one of the surgical procedures most often used for the treatment of spinal diseases [7, 27]. Spine fusion can be performed by several possible approaches, and one on the most employed is posterolateral spine fusion. Bone graft and pedicular screws are commonly used to improve fusion rates [28]. The former can be obtained from various sources, as has been mentioned in the introduction, but autologous graft is frequently preferred (either from the iliac crest or retrieved from the surgical site). Nevertheless, autologous iliac crest graft is associated to local pain and other complications. The graft retrieved locally may be insufficient to obtain spine fusion, and therefore, bone substitutes and substances with osteoconductivity, osteoinductive, or osteogenic capacity have been used [29]. Bone substitutes, such calcium phosphates, only have osteoconductive capacity and need other elements such as cells to have a comparable effect to that of autologous bone graft [30]. Osteoinductive molecules like morphogenetic proteins (BMPs) are expensive and have had associated problems with its use [31, 32].

In the last decade, cell therapy and tissue engineering have gained great interest in this setting. Numerous research studies have explored the potential of cell therapy for the treatment of various entities. MSCs have attracted more attention in the field of the skeletal diseases, due to the ability of these cells to differentiate into various mesodermal cells, such as bone or cartilage cell lineages [33, 34]. In addition to their ability to differentiate into various cell lines, these cells have an important immunomodulatory and anti-inflammatory capacities, which is very attractive for the treatment of some diseases [35, 36]. Moreover, these cells release to their surrounding cells many growth factors and exosomes that may favor bone regeneration and osteogenesis [37]. This may be even enhanced by the presence of TCP scaffolds [38]. These cells, described first by Friedenstein [39], have some characteristics that are necessary to prove, in order to consider that the cellular products fulfill MSC definition criteria [40]. This point is very important to know when reporting the results and effects of the use of these cells in different clinical situations. There is an extensive number of publications reporting results on the use of MSC for obtaining spinal fusion in small, medium, and large animal models [18]. But, there is also a number of studies where other cell types are used, including cells that are completely different to MSC, as the stromal vascular fraction (SVF). SVF, as opposed to MSC, are not included into the Advanced Therapies Medicinal Products (ATMP), and thus have a lighter regulation and can be done in an automated environment in the same surgical room [41]. Nevertheless, SVF have a mixture of different cell types, including some pro-inflammatory cells (as monocytes). In the same line, some other groups have used a bone marrow aspirate [42]. The bone marrow aspirate or concentrate is used in various combinations such as auto graft, human demineralized bone matrix, and biomaterials [43]. While it is true that BM aspirate or concentrate has beneficial effects in spinal fusion, the process to obtain it is simple and inexpensive [44–47], and it contains progenitor cells (mostly of hematopoietic origin and few MSC less than 0.1%), but their quantity and distribution can be variable [42, 43, 48]. These strategies, compared to Advanced Therapies Medicinal Products (ATMPs), may not provide a uniform cell product with exact characterization of the cells and proportions of defined subpopulations administered, as it occurs with International Society of Cellular Therapy (ISCT)-defined MSC [40], and are not considered a medicinal product regulated by the National Medicines Agencies.

Compared to previous works, our pilot study has some differential characteristics [18]. Firstly, as it has been just mentioned, it employs properly ISCT-defined MSC produced in a GMP facility with all the approvals of the corresponding regulatory agencies as an ATMP. The product is homogeneous and reproducible. Our trial, compared to a series of cases analyzed retrospectively, is a prospective study where autologous BM-derived MSCs were mixed with beta-tricalcium phosphate as a graft in posterolateral arthrodesis for the treatment of a degenerative disc disease-resistant to conservative treatment. Tricalcium phosphate has been employed in other studies [49, 50]. This bone substitute is easy to use and presents appropriate physical and chemical characteristics. It was used in our study, as it was the default bone substitute used at that time. Another characteristic of our study compared to others [42] is that the spine level where the treatment was applied is homogeneous, and therefore, the role of the treatment could be better compared in a phase I–II trial.

Our study also has some limitations, as the low number of patients included. As already mentioned, it was designed as a phase I/II trial. Although conclusions on efficacy of the procedure have to be taken cautiously, the results are comparable with the other approaches indicated before. In addition, we have not tested different cell doses, which would have an impact in improving these results, but this should be evaluated prospectively in future trials.

## Conclusions

We have demonstrated the safety and feasibility of the procedure and provided the longest follow-up published to date (more than 5 years for some patients, see Additional file 1), what further guarantees the achievement of these primary objectives of a phase I–II trial, where absence of secondary effects is the most important finding.

It is also important to note not only that the clinical situation of the patients improved (an 81% of fusion rate was achieved by the end of the trial), both for back pain and radicular pain, but also, all patients except one patient resumed their work activities.

In summary, our results show that cell therapy with autologous MSCs in posterolateral spinal fusion is a safe and feasible procedure, at least in our environment. Although the number of patients is low, the duration of follow-up was extended over 5 years.

## Additional file

Additional file 1: Supplemental information. Additional information of one patient long-term follow-up. (DOCX 337 kb)

## Abbreviations

ASA: American Society of Anesthesiologist
ATMP: Advanced Therapies Medicinal Products
BM: Bone marrow
BMP: Bone morphogenetic protein
DDD: Degenerative disc disease
ISCT: International Society of Cellular Therapy
MSC: Mesenchymal stromal cells
ODI: Oswestry disability index
SVF: Stromal vascular fraction
TCP: Tricalcium phosphate
VAS: Visual analog scale

## References

[1] Chan D, Song Y, Sham P, Cheung KM (2006). Genetics of disc degeneration. Eur Spine J.

[2] Setton LA, Chen J (2006). Mechanobiology of the intervertebral disc and relevance to disc degeneration. J Bone Jt Surg Am.

[3] Wang J, Tang T, Yang H, Yao X, Chen L, Liu W (2007). The expression of Fas ligand on normal and stabbed-disc cells in a rabbit model of intervertebral disc degeneration: a possible pathogenesis. J Neurosurg Spine.

[4] Bibby SR, Urban JP (2004). Effect of nutrient deprivation on the viability of intervertebral disc cells. Eur Spine J.

[5] Blanco JF, Graciani IF, Sanchez-Guijo FM, Muntión S, Hernandez-Campo P, Santamaria C (2010). Isolation and characterization of mesenchymal stromal cells from human degenerated nucleus pulposus: comparison with bone marrow mesenchymal stromal cells from the same subjects. Spine (Phila Pa 1976).

[6] Tarpada SP, Morris MT, Burton DA (2017). Spinal fusion surgery: a historical perspective. J Orthop.

[7] Rajaee SS, Bae HW, Kanim LE, Delamarter RB (2012). Spinal fusion in the United States: analysis of trends from 1998 to 2008. Spine (Phila Pa 1976).

[8] Rihn JA, Kirkpatrick K, Albert TJ. Graft options in posterolateral and posterior interbody lumbar fusion. Spine. 2005;35(17):1629–39. 10.1097/BRS.0b013e3181d25803 20628336

[9] Park JJ, Hershman SH, Kim YH (2013). Updates in the use of bone grafts in the lumbar spine. Bull Hosp Jt Dis.

[10] Dimitriou R, Mataliotakis GI, Angoules AG, Kanakaris NK, Giannoudis PV (2011). Complications following autologous bone graft harvesting from the iliac crest and using the RIA: a systematic review. Injury.

[11] An HS, Lynch K, Toth J (1995). Prospective comparison of autograft vs. allograft for adult posterolateral lumbar spine fusion: differences among freeze-dried, frozen, and mixed grafts. J Spinal Disord.

[12] Gibson S, McLeod I, Wardlaw D, Urbaniak S (2002). Allograft versus autograft in instrumented posterolateral lumbar spinal fusion: a randomized control trial. Spine (Phila Pa 1976).

[13] Korovessis P, Koureas G, Zacharatos S, Papazisis Z, Lambiris E (2005). Correlative radiological, self-assessment and clinical analysis of evolution in instrumented dorsal and lateral fusion for degenerative lumbar spine disease. Autograft versus coralline hydroxyapatite. Eur Spine J.

[14] Oryan A, Kamali A, Moshiri A, Baghaban Eslaminejad M (2017). Role of mesenchymal stem cells in bone regenerative medicine: what is the evidence?. Cells Tissues Organs.

[15] Uccelli A, de Rosbo NK (2015). The immunomodulatory function of mesenchymal stem cells: mode of action and pathways. Ann N Y Acad Sci.

[16] Sharma RR, Pollock K, Hubel A, McKenna D (2014). Mesenchymal stem or stromal cells: a review of clinical applications and manufacturing practices. Transfusion.

[17] Wang LT, Ting CH, Yen ML, Liu KJ, Sytwu HK, Wu KK (2016). Human mesenchymal stem cells (MSCs) for treatment towards immune- and inflammation-mediated diseases: review of current clinical trials. J Biomed Sci.

[18] Salamanna F, Sartori M, Brodano GB, Griffoni C, Martini L, Boriani S (2017). Mesenchymal stem cells for the treatment of spinal arthrodesis: from preclinical research to clinical scenario. Stem Cells Int.

[19] Blanco JF, García-Briñon J, Benito-Garzón L, Pescador D, Muntión S, Sánchez-Guijo F (2018). Human bone marrow mesenchymal stromal cells promote bone regeneration in a xenogeneic rabbit model: a preclinical study. Stem Cells Int.

[20] Pfirrmann CW, Metzdorf A, Zanetti M, Hodler J, Boos N (2001). Magnetic resonance classification of lumbar intervertebral disc degeneration. Spine (Phila Pa 1976).

[21] Sánchez-Guijo F, Caballero-Velázquez T, López-Villar O, Redondo A, Parody R, Martínez C (2014). Sequential third-party mesenchymal stromal cell therapy for refractory acute graft-versus-host disease. Biol Blood Marrow Transplant.

[22] Williamson A, Hoggart B (2005). Pain: a review of three commonly used pain rating scales. J Clin Nurs.

[23] Little DG, MacDonald D (1994). The use of the percentage change in Oswestry disability index score as an outcome measure in lumbar spinal surgery. Spine (Phila Pa 1976).

[24] Walsh TL, Hanscom B, Lurie JD, Weinstein JN (2003). Is a condition-specific instrument for patients with low Back pain/leg symptoms really necessary?. Spine (Phila Pa 1976).

[25] Sathiyakumar V, Molina CS, Thakore RV, Obremskey WT, Sethi MK (2015). ASA score as a predictor of 30-day perioperative readmission in patients with orthopaedic trauma injuries: an NSQIP analysis. J Orthop Trauma.

[26] Werner BC, Li XSF (2014). Stem cells in preclinical spine studies. spine J.

[27] Weiss AJ, Elixhauser A (2006). Trends in operating room procedures in U.S. Hospitals, 2001–2011: Statistical Brief #171. Healthcare Cost and Utilization Project (HCUP) Statistical Briefs.

[28] Wood GW, Boyd RJ, Carothers TA, Mansfield FL, Rechtine GR, Rozen MJ (1995). The effect of pedicle screw/plate fixation on lumbar/lumbosacral autogenous bone graft fusions in patients with degenerative disc disease. Spine (Phila Pa 1976).

[29] Tuchman A, Brodke DS, Youssef JA, Meisel HJ, Dettori JR, Park JB (2016). Iliac crest bone graft versus local autograft or allograft for lumbar spinal fusion: a systematic review. Glob Spine J.

[30] Buser Z, Brodke DS, Youssef JA, Meisel H-J, Myhre SL, Hashimoto R (2016). Synthetic bone graft versus autograft or allograft for spinal fusion: a systematic review. J Neurosurg Spine..

[31] Cahill KS, McCormick PC, Levi AD (2015). A comprehensive assessment of the risk of bone morphogenetic protein use in spinal fusion surgery and postoperative cancer diagnosis. J Neurosurg Spine..

[32] Singh K, Ahmadinia K, Park DK, Nandyala SV, Marquez-Lara A, Patel AA (2014). Complications of spinal fusion with utilization of bone morphogenetic protein. Spine (Phila Pa 1976).

[33] Samsonraj RM, Raghunath M, Nurcombe V, Hui JH, van Wijnen AJ, Cool SM (2017). Concise review: multifaceted characterization of human mesenchymal stem cells for use in regenerative medicine. Stem Cells Transl Med.

[34] Pescador D, Ibáñez-Fonseca A, Sánchez-Guijo F, Briñón JG, Arias FJ, Muntión S, et al. Regeneration of hyaline cartilage promoted by xenogeneic mesenchymal stromal cells embedded within elastin-like recombinamer-based bioactive hydrogels. J Mater Sci Mater Med. 2017;28(8) 10.1007/s10856-017-5928-1 28647792

[35] Blanco B, Herrero-Sánchez M del C, Rodríguez-Serrano C, García-Martínez ML, Blanco JF, Muntión S (2016). Immunomodulatory effects of bone marrow versus adipose tissue-derived mesenchymal stromal cells on NK cells: implications in the transplantation setting. Eur J Haematol.

[36] Valencia J, Blanco B, Yáñez R, Vázquez M, Herrero Sánchez C, Fernández-García M (2016). Comparative analysis of the immunomodulatory capacities of human bone marrow– and adipose tissue–derived mesenchymal stromal cells from the same donor. Cytotherapy.

[37] Li Y, Jin D, Xie W, Wen L, Chen W, Xu J (2018). Mesenchymal stem cells-derived exosomes: a possible therapeutic strategy for osteoporosis. Curr Stem Cell Res Ther.

[38] Zhang J, Liu X, Li H, Chen C, Hu B, Niu X (2016). Exosomes/tricalcium phosphate combination scaffolds can enhance bone regeneration by activating the PI3K/Akt signaling pathway. Stem Cell Res Ther.

[39] Friedenstein AJ, Petrakova KV, Kurolesova AI, Frolova GP (1968). Heterotopic of bone marrow. Analysis of precursor cells for osteogenic and hematopoietic tissues. Transplantation.

[40] Dominici M, Le Blanc K, Mueller I, Slaper-Cortenbach I, Marini F, Krause DS (2006). Minimal criteria for defining multipotent mesenchymal stromal cells. The International Society for Cellular Therapy position statement. Cytotherapy.

[41] Bora P, Majumdar AS (2017). Adipose tissue-derived stromal vascular fraction in regenerative medicine: a brief review on biology and translation. Stem Cell Res Ther.

[42] Yousef MAA, La Maida GA, Misaggi B (2017). Long-term radiological and clinical outcomes after using bone marrow mesenchymal stem cells concentrate obtained with selective retention cell technology in posterolateral spinal fusion. Spine (Phila Pa 1976).

[43] Gan Y, Dai K, Zhang P, Tang T, Zhu Z, Lu J (2008). The clinical use of enriched bone marrow stem cells combined with porous beta-tricalcium phosphate in posterior spinal fusion. Biomaterials.

[44] Hendrich C, Engelmaier F, Waertel G, Krebs R, Jäger M (2009). Safety of autologous bone marrow aspiration concentrate transplantation: initial experiences in 101 patients. Orthop Rev (Pavia)..

[45] Jäger M, Hernigou P, Zilkens C, Herten M, Li X, Fischer J, et al. Cell therapy in bone healing disorders. Orthop Rev (Pavia). 2010;2(2):e20. 10.4081/or.2010.e20 PMC3143975 21808710

[46] Jäger M, Herten M, Fochtmann U, Fischer J, Hernigou P, Zilkens C (2011). Bridging the gap: bone marrow aspiration concentrate reduces autologous bone grafting in osseous defects. J Orthop Res.

[47] Gessmann J, Köller M, Godry H, Schildhauer TA, Seybold D (2012). Regenerate augmentation with bone marrow concentrate after traumatic bone loss. Orthop Rev (Pavia).

[48] Hyer CF, Berlet GC, Bussewitz BW, Hankins T, Ziegler HL, Philbin TM (2013). Quantitative assessment of the yield of osteoblastic connective tissue progenitors in bone marrow aspirate from the iliac crest, tibia, and calcaneus. J Bone Joint Surg-Am.

[49] Epstein NE (2009). Beta tricalcium phosphate: observation of use in 100 posterolateral lumbar instrumented fusions. Spine J.

[50] Thaler M, Lechner R, Gstottner M, Kobel C, Bach C (2013). The use of beta-tricalcium phosphate and bone marrow aspirate as a bone graft substitute in posterior lumbar interbody fusion. Eur Spine J.

