# Supplementary material for: Autologous mesenchymal stromal cells embedded in tricalcium phosphate for posterolateral spinal fusion: results of a prospective phase I/II clinical trial with long-term follow-up
Source: Stem Cell Res Ther. 2019 Feb 22;10:63. doi: 10.1186/s13287-019-1166-4 (PMC6387529; doi:10.1186/s13287-019-1166-4)
Supplement: Supplementary file 1 — Supplemental information. Additional information of one patient long-term follow-up. (DOCX 337 kb) [file 13287_2019_1166_MOESM1_ESM.docx]

# Supplemental information

Patient number 5, four years after having completed the follow-up, consulted due to severe low-back pain of a mechanical nature. The neurological examination was normal. Image through x-ray and MRI studies confirmed the existence of a deterioration of a contiguous disc level. In the absence of response to the conservative treatment, surgery for fusion with extension to the adjacent level was performed. Posterolateral bone fusion of the index procedure was evidenced during the procedure. A sample from both sides was collected and histological study was performed. The findings were similar on both sides, showing mature bone (Additional Image 1).


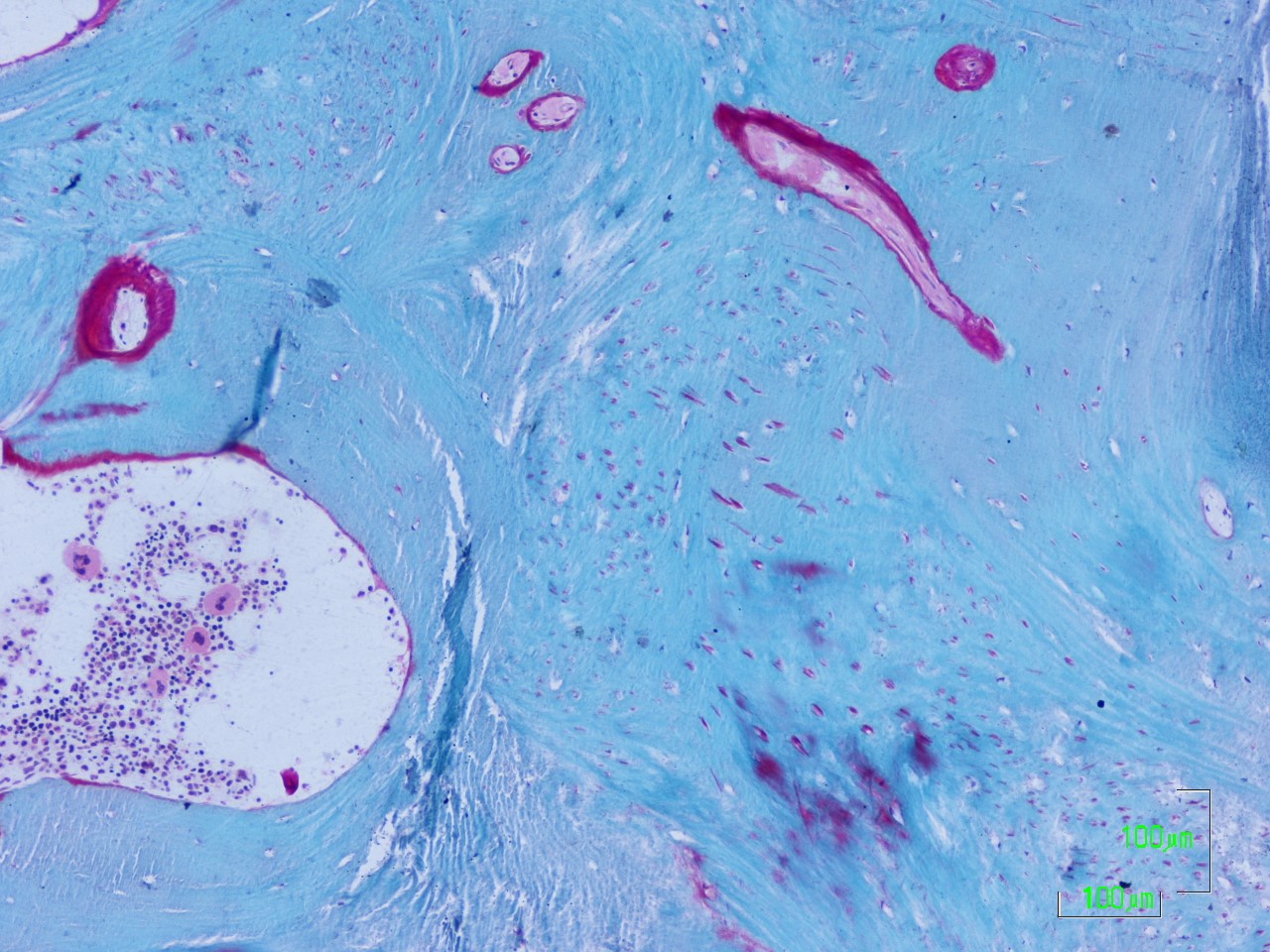


Additional Image 1. Histological Image from Patient #5 demonstrating mature bone formation.
